# Liquid-phase mega-electron-volt ultrafast electron diffraction

**DOI:** 10.1063/1.5144518

**Published:** 2020-03-09

**Authors:** J. P. F. Nunes, K. Ledbetter, M. Lin, M. Kozina, D. P. DePonte, E. Biasin, M. Centurion, C. J. Crissman, M. Dunning, S. Guillet, K. Jobe, Y. Liu, M. Mo, X. Shen, R. Sublett, S. Weathersby, C. Yoneda, T. J. A. Wolf, J. Yang, A. A. Cordones, X. J. Wang

**Affiliations:** 1 Department of Physics and Astronomy, University of Nebraska-Lincoln, Lincoln, Nebraska 68588, USA; 2 Department of Physics, Stanford University, Stanford, California 94305, USA; 3 Stanford PULSE Institute, SLAC National Accelerator Laboratory, Menlo Park, California 94025, USA; 4 SLAC National Accelerator Laboratory, Menlo Park, California 94025, USA; 5 Department of Applied Physics, Stanford University, Stanford, California 94305, USA; 6 Department of Physics and Astronomy, Stony Brook University, Stony Brook, New York 11794, USA

## Abstract

The conversion of light into usable chemical and mechanical energy is pivotal to several biological and chemical processes, many of which occur in solution. To understand the structure–function relationships mediating these processes, a technique with high spatial and temporal resolutions is required. Here, we report on the design and commissioning of a liquid-phase mega-electron-volt (MeV) ultrafast electron diffraction instrument for the study of structural dynamics in solution. Limitations posed by the shallow penetration depth of electrons and the resulting information loss due to multiple scattering and the technical challenge of delivering liquids to vacuum were overcome through the use of MeV electrons and a gas-accelerated thin liquid sheet jet. To demonstrate the capabilities of this instrument, the structure of water and its network were resolved up to the 3rd hydration shell with a spatial resolution of 0.6 Å; preliminary time-resolved experiments demonstrated a temporal resolution of 200 fs.

## I. INTRODUCTION

Ultrafast solution phase photochemistry is the pillar of many biological and chemical processes, such as vision, photosynthesis, and DNA photodamage,^1–3^ responsible for converting light into usable chemical and mechanical energy. The atomistic understanding of these chemical processes requires characterization of both solute and solvent dynamics, as the reaction environment can dictate rates, pathways, and efficiencies of reactions. Many spectroscopic methods have been developed to probe reaction dynamics in solution. However, these are not directly sensitive to the position of the nuclei and often infer the nuclear structure from changes in the valence electronic structure.^4^ Time-resolved scattering techniques, on the other hand, offer direct access to structural information concerning all atom pairs in a solution, allowing the simultaneous capture of solute, solvent, and solute-solvent interaction dynamics. Time-resolved diffuse x-ray scattering experiments have been successfully used to track structural changes in a variety of solution-phase systems at both the picosecond^5^ and femtosecond^6^ time scales. The shallow penetration depth of electrons (typically <1 *μ*m, even at MeV energies^7^) compared to hard x rays (typically >100 *μ*m) had, until recently, limited their use in the study of liquid-phase samples, as excessive multiple scattering prevents the retrieval of structural information. Previous attempts at using electrons to probe the structure of liquids had relied on slowly evaporating films,^8^ thin layer vapor deposition,^9,10^ and liquid cells^11^ to generate thin samples. However, a rapidly refreshed sample is required for ultrafast electron diffraction (UED) measurements, as such, the sample delivery method must allow liquid flow. Liquid jets^12^ and nanofluidic flow cells^13,14^ show promise in offering thin, flowing samples; here, we adopt the thin liquid jet approach developed by DePonte *et al.*, which showed great promise in static scattering experiments. Jet characterization via transmission electron microscopy showed elastic scattering to overcome the inelastic background at jet thicknesses below 800 nm, motivating the development of thinner gas-accelerated liquid sheet jets.^12^

The liquid-phase ultrafast electron diffraction (LUED) instrument presented here minimizes the loss of information due to multiple scattering through the use of mega-electron-volt (MeV) electrons and a gas-accelerated liquid sheet jet^12,15^ capable of producing sample thicknesses on the order of 100 nm. The use of relativistic electrons^16,17^ not only overcomes the temporal resolution penalty associated with the velocity mismatch between the pump laser and electron probe but also reduces the space-charge induced broadening of electron bunches. This allows the liquid jet, typically held at 10^−4 ^Torr, to be far from the electron source, held at ultra-high vacuum, while still preserving a temporal resolution of sub-200 fs. The use of a continuous flow gas-accelerated liquid jet ensures that the sample volume is refreshed after every shot at sample thicknesses amenable to scattering experiments using MeV electrons. Preliminary studies of liquid water using the MeV LUED instrument show spatial and temporal resolutions identical to those reported in MeV UED studies in the gas phase.^18–20^ Moreover, liquid-phase UED holds the promise of providing structural information similar to, and in some cases, complementary to, that obtained from x-ray scattering experiments. The sensitivity of electrons to the total charge distribution (Coulomb potential) of the sample^21^ may be of particular importance in the study of photochemical mechanisms mediated by electron and/or proton transfer events. In combination with the ability to achieve both sub-200 fs full width at half maximum (FWHM) temporal resolution and momentum transfer ranges in excess of 10 Å^−1^, this makes liquid-phase MeV UED a method to probe solution phase photochemistry with the potential to resolve effects, e.g., from hydrogen bonding.

**FIG. 1.**
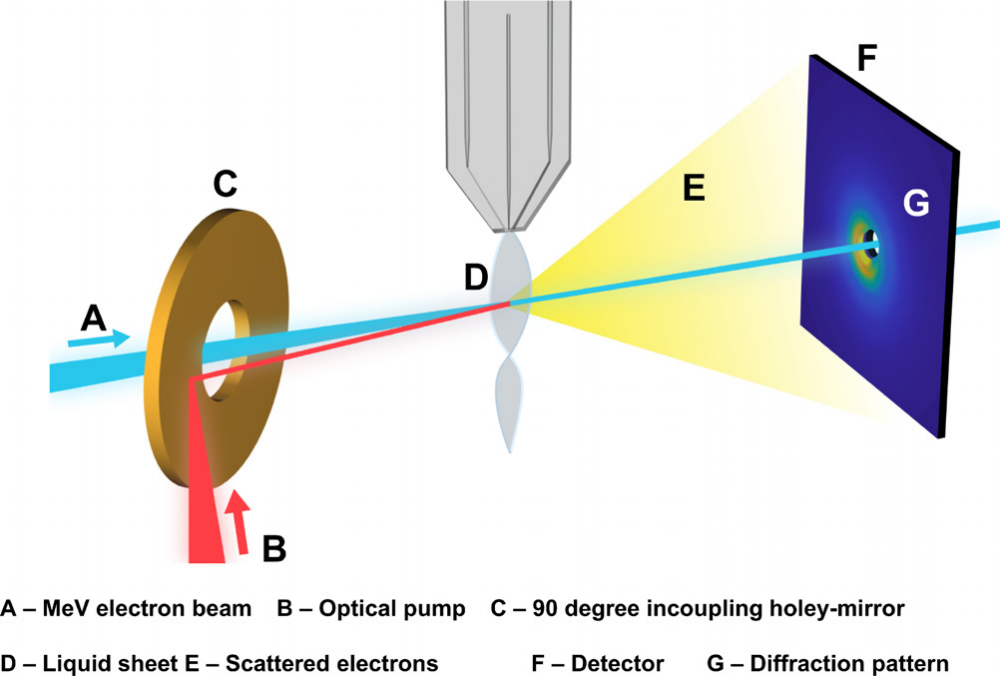
Schematic representation of the liquid-phase MeV UED experimental setup, illustrating a MeV electron beam (A) traversing a thin liquid sheet (D) and the scattered electron (E) being recorded as a diffraction pattern (G) at the detector (F). Species in the liquid sheet are excited by an optical pump (B) made to travel colinearly to the electron beam by a 90° holey-mirror (C). A detailed description of our MeV LUED instrument can be found in Sec. IV.

In this manuscript, we report on the design and commissioning of an MeV LUED instrument for optical pump-electron probe studies of liquid-phase samples, enabling the use of the MeV UED in liquid samples for the first time. The MeV LUED instrument, depicted schematically in Fig. 1, uses an MeV electron beam to probe the structure of molecules in a thin liquid sheet produced by a gas-accelerated liquid jet. Electrons scattered by the 100 nm thick liquid sheet are detected as diffraction patterns several meters downstream of the interaction point. In Sec. II, we present commissioning results that demonstrate the spatial and temporal resolution of the LUED instrument as well as the properties of the liquid jet. In particular, we report on the static diffraction signal for liquid water and its plasma lensing response upon illumination with 800 nm light. An outlook onto the future development of the technique is presented in Sec. III. The design of the instrument is presented in Sec. IV in three parts: (A) Integration with the SLAC MeV UED beamline, (B) Sample chamber, and (C) Sample delivery.

## II. RESULTS

### A. Static electron diffraction of pure water

**FIG. 2.**
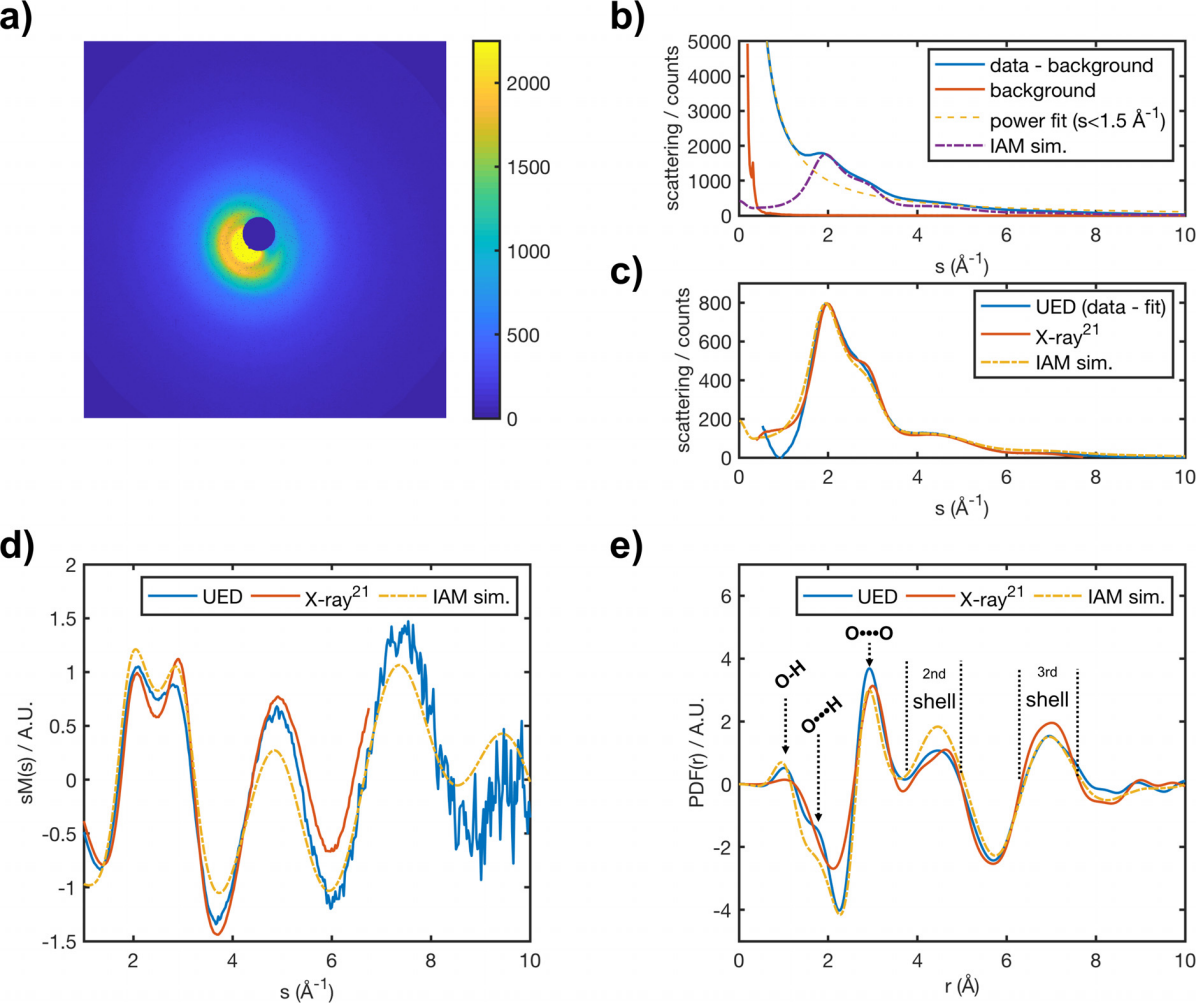
Panel (a) shows an average diffraction pattern and panel (b) the azimuthally averaged scattering signal for liquid water at 290 K, acquired over 350 s. The temperature of the water was determined using the method described in Sec. II C. The background signal (orange line) in panel (b) was acquired by switching off the flow of water and maintaining a constant flow of helium. Panels (c) and (d) show UED, x-ray and simulated scattering curves, and *sM*(*s*) curves for liquid water, respectively. The simulated *sM*(*s*) is generated under the independent atom model (IAM) approximation and assuming a water temperature of 290 K. Residual experimental background response contributions were subtracted from both the UED and x-ray scattering curves [panel (c)] using a third order polynomial curve fitted over the entire *s* range to obtain *sM*(*s*) curves [panel (d)]. The x-ray scattering data were measured for liquid water at the European Synchrotron Radiation Facility, using the conditions described in Ref. 22. Panel (e) shows UED, x-ray, and simulated *pdf*(*r*) curves for liquid water.

Electron diffraction for liquid water was acquired as part of the LUED instrument commissioning. In Figs. 2(a) and 2(b), we present an average diffraction pattern and the corresponding azimuthally averaged scattering signal for static water, integrated over 350 s and expressed as a function of momentum transfer, *s*,

s=(4π/λ) sin (θ/2), (1)

where *λ* is the de Broglie wavelength of the electron, 0.3 pm in the case of our 3.7 MeV electron beam, and *θ* is the angle between scattered and unscattered electrons. The experimentally available momentum transfer range is *s *=* *0.4–10 Å^−1^; based on the inverse relationship between *s* and real-space distances, the spatial resolution is $2\pi /s_{\text{max}}=0.6$ Å.

A method analogous to those employed in the analysis of gas electron diffraction (GED)^23^ is here employed in the retrieval of structural information from the electron diffraction pattern of liquid water. Assuming elastic scattering, the total scattering intensity, *I*(*s*), for a sample of randomly oriented molecules can be expressed as the sum of the atomic, $I_{at}(s)$, and molecular, $I_{\mathrm{mol}}(s)$, scattering terms: *I*(*s*) = $I_{at}(s)$ + $I_{\mathrm{mol}}(s)$. The contribution of the atomic scattering to the overall scattering intensity is simply given as the sum of all the elastic scattering amplitudes for all atoms in the system,

$$I_{\text{at}}(s)=\sum_{i=1}^{N}{\left|f_{i}(s)\right|}^{2},$$ (2)

where *N* is the number of atoms in the system and $f_{i}(s)$ is the elastic scattering amplitude for the *i*th atom. The elastic scattering amplitude for an MeV electron can be calculated using the ELSEPA program.^24^ Note that $I_{at}(s)$ [Eq. (2)] does not contain structural information. The molecular scattering term, on the other hand, can be expressed as a sum of interference terms for all atom pairs in the system,

$$I_{\text{mol}}(s)=\sum_{i=1}^{N}\sum_{j\neq 1}^{N}\left|f_{i}(s)\right|\left|f_{j}(s)\right|\;\text{cos}\;(\eta_{i}-\eta_{j})\frac{\;\text{sin}\;(sr_{ij})}{sr_{ij}},$$ (3)

where $f_{i}(s)$ and $f_{j}(s)$ are the elastic scattering amplitudes of the *i*th and *j*th atom, respectively, and $\eta_{i}(s)$ and $\eta_{j}(s)$ are their corresponding phases. Both elastic scattering amplitudes and phases are calculated using the ELSEPA package.^24^ *r_ij_* is the internuclear separation between the *i*th and *j*th atoms.

Sinusoidal modulations imparted to the scattering intensity by the interference term are made clearer through the use of a modified scattering intensity, *sM*(*s*), defined as

$$sM(s)=\frac{I_{\text{mol}}(s)}{I_{\text{at}}(s)}s.$$ (4)

The experimental modified scattering intensity for liquid water was calculated using an adaptation of the method developed by Ihee *et al.*,^25^ in which *sM*(*s*) is expressed as

$$sM(s)=\frac{I_{\;\text{exp}\;}-I_{\text{bkg}}}{I_{\text{at}}(s)}s,$$ (5)

where $I_{\;\text{exp}}$ is the experimental scattering intensity and $I_{\text{bkg}}$ is a smooth experimental background response, which includes elastic atomic scattering, inelastic scattering, and system-specific background. *I_at_* is the theoretical atomic scattering intensity for the sample [see Eq. (2)]. In the case of our water diffraction, the experimental background response contribution to the total scattering can be approximated by fitting a smooth power curve ($I_{\text{bkg}}=As^{n}$) to $I_{\;\text{exp}}$.

Theoretical *sM*(*s*) curves for liquid water were determined with the aid of molecular dynamics (MD) simulations and a method developed by Dohn *et al.*,^26^ which allows the calculation of theoretical scattering intensities from MD generated pairwise radial distribution functions. Under the independent atom model (IAM) approximation, the atomic and molecular elastic scattering intensities of a system in the liquid phase are given by

$$I_{\text{at}}(s)=\sum_{l}N_{l}{\left|f_{l}(s)\right|}^{2},$$ (6)

$$I_{\text{mol}}(s)=\sum_{l,m}\left|f_{m}(s)\right|\left|f_{l}(s)\right|\frac{N_{m}(N_{l}-\delta_{m,l})}{V}4\pi \int_{0}^{R}r^{2}g_{l,m}(r)\frac{\;\text{sin}\;(sr)}{sr}dr,$$ (7)

where *N_l_* and *N_m_* are the number of occurrences of atom types *l* and *m*, which in our application correspond to different elements. Similarly, $f_{m}(s)$ and $f_{l}(s)$ correspond to the elastic scattering amplitude of elements *l* and *m*. $g_{l,m}$ is the pair radial distribution function for the *l* and *m* elements, $\delta_{m,l}$ is the Kronecker delta, and *R* is the radius of the coherence volume, *V*, in the sample. The derivation of Eqs. (6) and (7) can be found in Ref. 26. Classical MD simulations of 4054 water molecules (50×50×50 Å box) were carried out using the TIP4P-Ew force-field^27^ at constant temperature and pressure. TIP4P-Ew is a good general-purpose model for water, purposely tuned to reproduce the bulk-density and enthalpy of vaporization of liquid water^28^ and with predicted structural properties (O–O radial distribution functions) in good agreement with those observed using x-ray scattering.^27,29^ The MD simulations were carried out over 1000 ps in 2 fs time steps using the GROMACS package.^30^ The resulting MD trajectories were processed into time averaged pairwise radial distribution functions using the VMD package.^31^ The temperature of the MD simulations was varied between 250 and 400 K using the Berendsen thermostat^32^ and using Eqs. (6) and (7), a theoretical scattering curve was generated for each temperature. As the position of the first diffraction peak of water is strongly dependent on the temperature of the water,^33^ the position of the first peak in our experimental data was used to determine the temperature of the sample and select the adequate simulation temperature. Using this method, the experimental data presented in this section were determined to correspond to water at 290 K. A more detailed description of the sample temperature determination method can be found in Sec. II C. The experimental and simulated modified scattering intensities for liquid water at 290 K are presented in Fig. 2(d). The sine transform of the modified scattering intensity can be used to retrieve structural information in the form of a pair distribution function, *pdf*(*r*),

$$\mathrm{pdf}(r)=\int_{0}^{s_{\text{max}}}sM(s)\;\text{sin}\;(sr)\;\text{exp}\;(-ks^{2})ds,$$ (8)

where *k* is a damping factor used to suppress high frequency artifacts generated by the truncation of *sM*(*s*) at s= 10 Å^−1^.

The experimental and simulated pair distribution functions for liquid water are shown in Fig. 2(e). The positions of features in the *pdf*(*r*), corresponding to ensembles of similar internuclear distances, match those predicted by our IAM simulations and observed in x-ray scattering experiments.^34–37^ Therefore, we are able to assign these features to the structural motifs of liquid water. The peak at 1.0 and shoulder at 1.8 Å correspond to the bonded O–H and hydrogen bonded O ⋯ H internuclear distances in the water's first hydration shell, respectively. The ability to resolve the hydrogen bonded O ⋯ H is of particular importance, given the lack of sensitivity of x rays to hydrogen nuclei. The lack of electron density around the nucleus of a bonded hydrogen atom results in a rather weak signal, not detectable in most cases. The peak at 2.9 Å corresponds to the non-bonded O ⋯ O internuclear distances between two neighboring waters, while the broad peaks centered at 4.4 and 6.9 Å correspond to internuclear distances across the 2nd and 3rd hydration shell of water, respectively.

To ensure that our comparison of UED and x-ray *pdf*(*r*) remained unbiased from theoretical input, we relied on rather simple power and polynomial fits to remove the experimental background response. As a result, instances in which the background response cannot be adequately modeled by either a power or low order polynomial functions lead to background contributions to the *pdf*(*r*). These are believed to be the source of the discrepancies between the experimental and simulated *pdf*(*r*) amplitudes. On the other hand, discrepancy between experimental and simulated total scattering, most noticeable in the low *s* region [Fig. 2(b)], has been attributed to the breakdown of the independent atom model and will be discussed in a follow up publication.

### B. Temporal resolution

In the absence of fast features with a known temporal profile, only the upper limit of the time resolution can be estimated, from the full width at half maximum (FWHM) of the fastest feature observed in time-resolved experiments. We estimate the time resolution from the duration of the low-*s* difference signal resulting from plasma lensing of the electron beam by the laser-ionized sample, as used in gas-phase UED to find temporal overlap.^38^

**FIG. 3.**
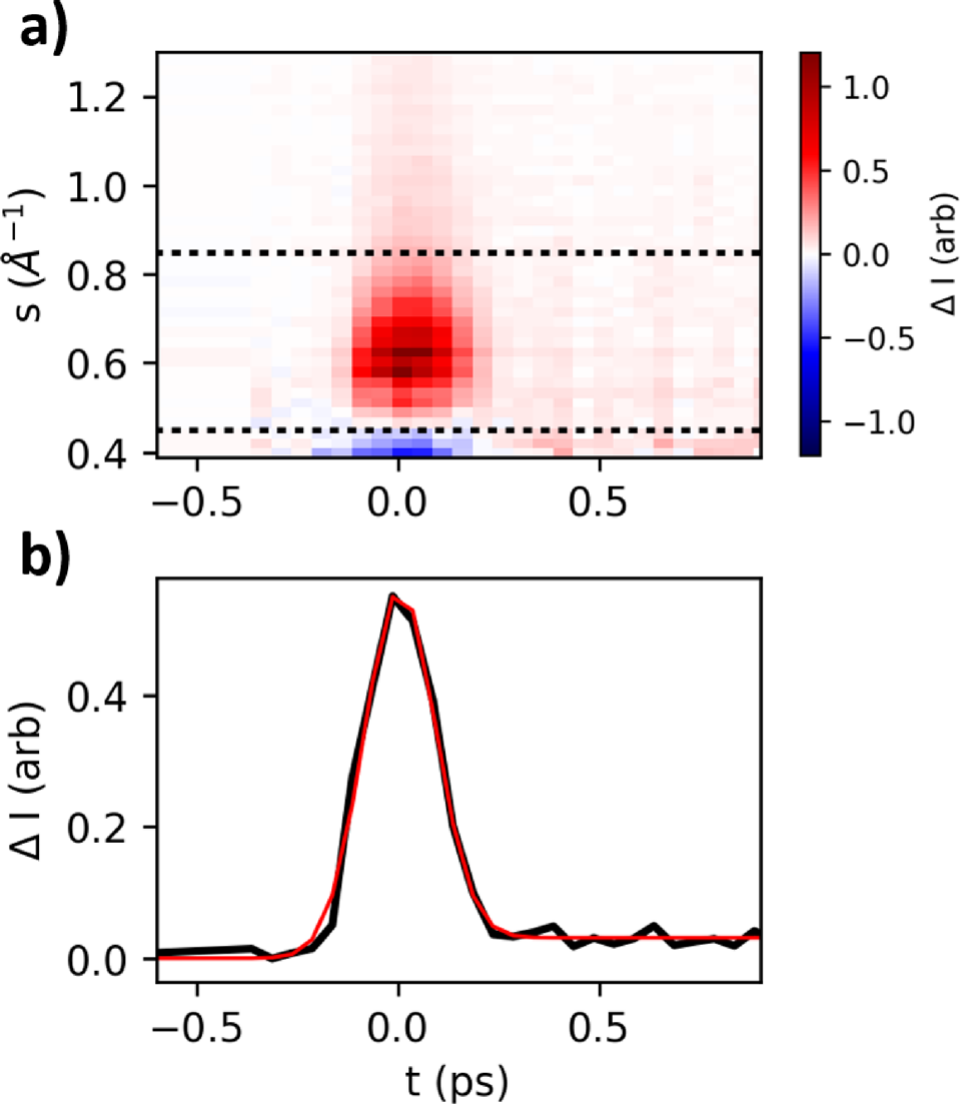
Upper-limit time resolution estimated from a low-*s* beam streaking effect in water excited at 800 nm, 1.1 J/cm^2^ fluence. (a) Difference scattering signal ΔS as a function of time delay and *s*, averaged over six 23-min scans. The negative signal at the lowest *s* and positive signal up to 1 Å^−1^ are a result of the main beam profile becoming elongated when passing through the ionized sample. (b) Time trace (black) of ΔS integrated between the dotted lines in panel (a), and fit (red). The FWHM of the feature is 209 ± 4 fs.

In pure water pumped at 800 nm, a fast feature is observed in the difference scattering integrated from *s *=* *0.45 to 0.85 Å^−1^. The low-*s* region of difference scattering is shown in Fig. 3. The signal was fit to the sum of a Gaussian, representing the plasma lensing signal, and a step function convolved with the same Gaussian, representing the small structural signal, which appears after *t*_0_. The FWHM of the Gaussian is used as an upper-limit estimate of the experimental time resolution. For a laser pump pulse fluence of 1.1 J/cm^2^ (64 fs FWHM pulse duration measured before one lens and window), the signal is visible after a single scan (9000 shots per pump-probe delay, 45 delays, 23 min of lab time). During stable operation of the electron gun, the minimum single-scan FWHM of this feature was 180 ± 20 fs. When averaged over six scans, there was some broadening to 209 ± 4 fs, due to a slow drift of temporal overlap by 60 fs over 4 h of lab time.

The pump laser in this experiment was incident at a 30° angle with respect to the electron beam, which introduced additional time smearing. For a pump pulse spot size of ≈ 50 *μ*m (smaller than the electron beam), the tilted incidence is expected to broaden the time resolution by 30 fs. The overall time resolution performance in the liquid phase is therefore comparable to that of gas phase experiments at the facility, which reported time resolutions of 160^19^ to 230^38^ fs.

### C. Jet parameters

**FIG. 4.**
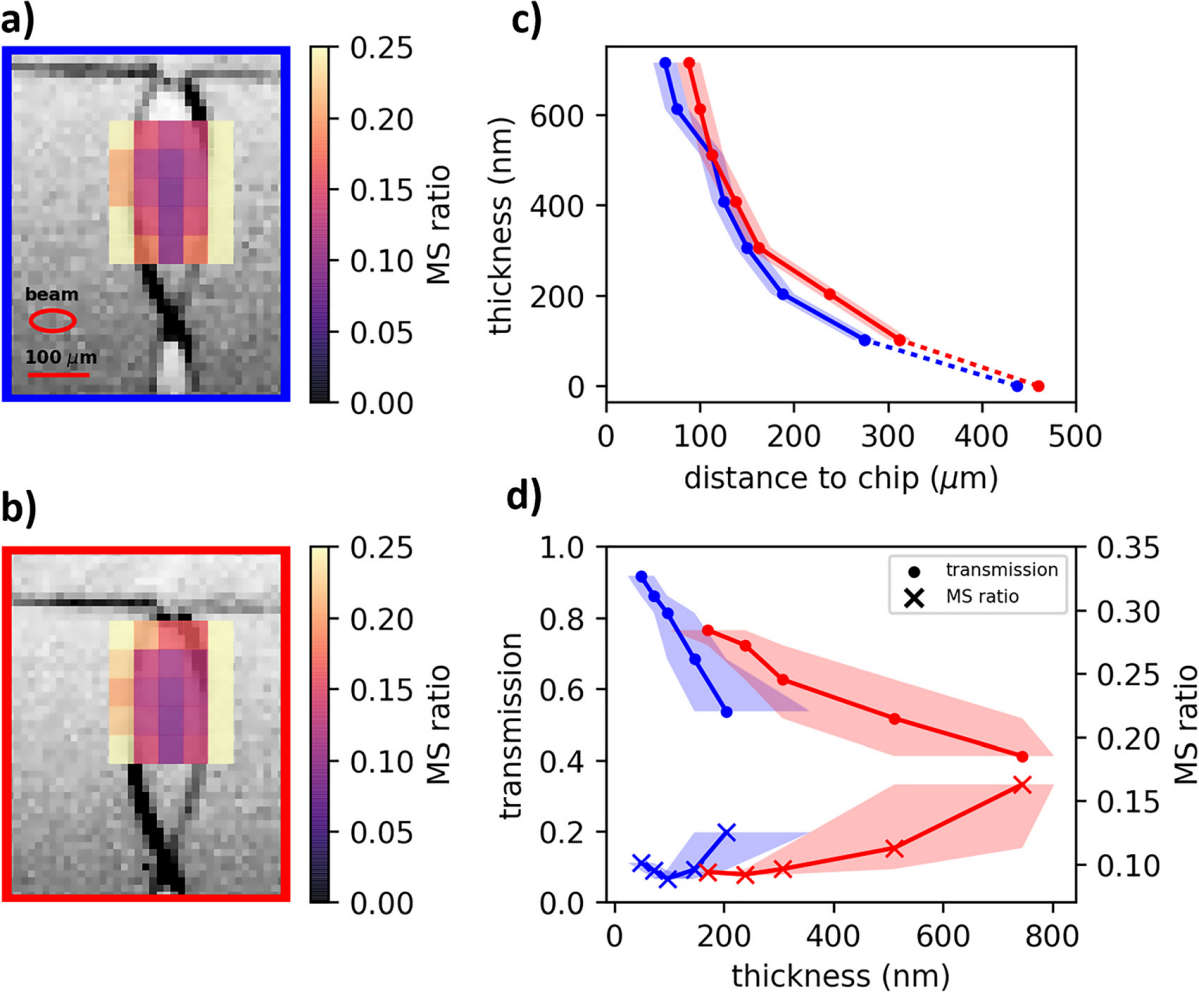
(a) and (b) Images taken at 30° from normal of liquid jet at 0.20 and 0.25 ml/min liquid flow rate, respectively, and equal He pressure; liquid flow is from top to bottom. Overlay: multiple scattering ratio, as defined in text, for a grid of electron beam positions. The scale bar is 100 *μ*m; the FWHM electron beam size is also shown. (c) Thickness of jets (a) (blue) and (b) (red) as a function of distance from the nozzle, as measured by thin-film interference. Error bars represent 12.5 *μ*m resolution of camera; the dotted line represents region where the jet is thinner than the sensitivity of the interference measurement (102 nm) as described in Sec. IV B 4. (d) Transmission and multiple scattering ratio at the center of the jet as a function of jet thickness for the two jet conditions. The shaded region corresponds to ±50 *μ*m uncertainty in the vertical position of electrons on the jet.

The sample delivery for liquid-phase UED is bound by the constraint that the thickness of the sample must be less than 20% of the electron mean free path,^39^ which is about 1 *μ*m in water for 3.7 MeV electrons, to avoid significant multiple scattering of electrons within the sample and determine coordination numbers to an accuracy better than 10%.^39^ Therefore, the sample delivery system is designed around a gas-accelerated ultrathin sheet jet, which is able to deliver sub-100 nm liquid sheets.^15^ Using a glass microfluidic chip, the initially cylindrical liquid jet, 20 *μ*m in diameter, is flattened from either side by gas flow, thus producing a sub-micrometer sheet. The variable liquid flow rate and gas pressure allow the dimensions of the liquid sheet to be tuned. A range of pure water sheet parameters, from 0.15 to 0.25 ml/min liquid flow rate and 65–78 psi helium, were characterized to determine optimal jet parameters. Images taken under two of these conditions, 0.20 and 0.25 ml/min water flow and 78 psi helium, are shown in Fig. 4, panels (a) and (b). These conditions produced the largest liquid sheets, 425–450 *μ*m in length and 140–160 *μ*m in width. The thickness of the sheets, as measured via the interferometric imaging described in Sec. IV B 4, is reported in Fig. 4(c) as a function of distance from the nozzle. The measured thickness profile decreases from 700 nm near the top of the jet to sub-100 nm at the bottom. The increase in the flow rate from jet **a** to **b** manifests as a wider and longer, but also thicker, liquid sheet. The gas pressure was set to the highest possible pressure that still allowed for stable jet operations (<80 psi).

To assess the best UED measurement conditions, the liquid sheet was characterized via static electron scattering at a grid of points spaced by 50 *μ*m in the x and y directions. Two parameters were extracted from these scattering data. First, the transmission of electrons through the sheet was measured by a second detector behind the hole in the main detector phosphor screen (see Sec. IV A), which measured the non-scattered beam. The transmitted electron counts were divided by average counts on the detector when no jet was present. The average count rate without the jet was considered a constant between the two conditions shown, though changes in the background pressure in the chamber could introduce uncertainty in comparing absolute transmission values between the two conditions. Transmission as high as 92% was measured for the thinnest part of the sheet [Fig. 4(d)]. The second parameter was the ratio of high- to low-*s* scattering signal, which will be referred to as the multiple scattering ratio. Singly scattered electrons are expected to scatter most strongly around 2 Å^−1^ (the liquid peak). Multiply scattered electrons appear at all momentum transfers, creating a background throughout the scattering pattern. Therefore, we use the ratio of scattering integrated between *s *=* *6.8–9.0 Å^−1^ and *s *=* *1.6–3.1 Å^−1^ as a relative measure of the amount of multiple scattering present in an image. A low ratio corresponds to relatively little multiple scattering, and therefore a lower-background measurement of the elastic single scattering. This ratio at each of the 25 grid points is shown as an overlay on the jet images in Fig. 4, with a 50 *μ*m uncertainty in the vertical position of the electron probe relative to the jet image. The value of the ratio along the center of the sheet is shown in panel (d).

The transmission of electrons monotonically follows the measured sheet thickness; however, the multiple scattering ratio exhibits a minimum as the electrons are scanned vertically down the center of the sheet. This effect can be attributed to scattering from the sheet edges [see overlay in Figs. 3(a) and 3(b)]. While the sheet is thin in the center, the edges are much thicker than the sheet itself, estimated at 10 *μ*m. A small fraction of the electron beam scattering in the sheet edge will not strongly affect the transmission. However, since the thickness at the edge is many times greater than the electron mean free path, all electrons incident on the edge can be expected to scatter multiple times and contribute to the uniform background on the scattering detector. In the lower part of the sheet, the multiple scattering background increases due to clipping the electron beam on the edges of the jet.

Therefore, optimal measurement conditions are achieved at the vertical midpoint of the sheet. The lower portion of the jet, although thinner, cannot be used due to the prevalence of multiple scattering induced by the thick sheet edges. Comparable signal quality could be achieved with the higher flow rate jet, despite the sheet being thicker by a factor of two, because the greater width allowed the whole electron beam to pass through the sheet. The transmission of electrons alone, which is not as sensitive to the jet edges, is not a sufficient figure of merit to determine optimal beam placement on the jet.

**FIG. 5.**
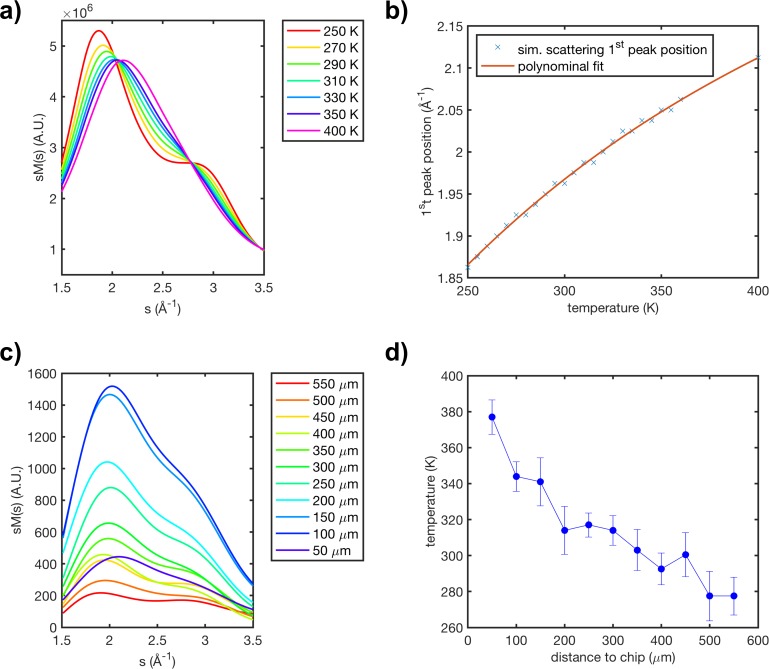
Panel (a) shows the simulated scattering signal of water over the 1.5–4 Å^−1^ range for temperatures ranging between 250 and 400 K. Panel (b) shows the evolution of the first peak position in the scattering signal of water as a function of temperature. Panel (c) shows the first peak of water scattering data acquired at varying distances from the chip. Panel (d) shows the estimated water temperature as a function of distance to the chip.

The temperature gradient across the liquid sheet was characterized using the static diffraction of water. First, the scattering signal of water was simulated for temperatures ranging between 250 and 400 K, using the method described in Sec. II. This revealed a strong dependency between the position first diffraction peak and the temperature of the water, with lower temperatures resulting in the shifting of the first diffraction peak toward lower momentum transfer *s* values, as shown in panel (a) of Fig. 5. This trend agrees with a previous experiment by x-ray scattering.^33^ The evolution of the first diffraction peak position as a function of temperature was fitted with a third order polynomial, resulting in the calibration curves shown in panel (b) of Fig. 5. A series of diffraction patterns were recorded at different points along the liquid sheet and the position of the first scattering peak was extracted and compared to the calibration curve to produce a temperature estimate. Sheet temperature estimates generated from this comparison are shown in panel (d) of Fig. 5. The top of the sheet, nearest the chip, is considerably warmer than room temperature due to radiative heating of the chip and chip holder by the heated catcher. The temperature decreases down the sheet, as the liquid undergoes rapid evaporative cooling. While cooling occurs in all jet conditions, the absolute temperature values are expected to depend heavily on the distance between the catcher and chip, the solvent used, and the thickness of the jet. Considering the 13 m/s speed of the jet, the temperature gradient implies cooling on the order of 10^6^ K/s. This is roughly an order of magnitude faster than reported values for supercooled water in 5 *μ*m cylindrical jets^40^ and two orders of magnitude faster than in 12 *μ*m droplets.^33,41^

Jet stability cannot be evaluated on a shot-by-shot basis due to the integrating mode of the detector (generally 5 s per image). However, image-to-image stability on the time scale of minutes to hours can be addressed. The intensity of the transmitted beam without a sample, measured by the secondary detector, has 6% rms fluctuations over 2.3 min of lab time. In comparison, the beam transmitted through the jet exhibits 7% rms fluctuations on the same time scale. However, on longer timescales, slow changes in the transmission (corresponding to the thickness of the jet) can be observed, with a 19% decrease in transmission over 4 h observed in a pure water jet.

The microfluidic chips were used continuously for as long as 40 h before replacement, with 15 h of data collection on pure water being typical before the chip required replacement. Pure solvents give the most stable jet start/stop and running conditions. However, highly concentrated aqueous solutions were also successfully run in the LUED chamber. Ionic solutes at 100 mM and 500 mM concentration ran for a maximum of 16 h without interruption (typical run time 8 h), despite some buildup of salt on the catcher cone and jet nozzle. Sample chamber running pressures with solutes were as low as for pure water jets, which ranged from 8 × 10^−5^ to 2 × 10^−4 ^Torr.

The gas-accelerated sheet jets, while designed for water jets, can also deliver other solvents. Ethanol jets were demonstrated in vacuum with a sample chamber pressure of 2 × 10^−4 ^Torr. Due to its higher vapor pressure, ethanol requires the collection bottle, as described in Sec. IV C, to be held at −20 °C. Sample-specific testing is necessary to determine whether solute deposition on the nozzle, due to rapid evaporation, will destabilize the jet in vacuum.

The chip holder can accept different types of microfluidic chips and is not limited to gas-accelerated nozzles. Converging-type^42^ nozzles, which create liquid sheets without gas flow, were also used in the LUED chamber. These nozzles create larger sheets with width and length dependent on the liquid flow rate. Typical sizes of 2 mm in length and 0.5 mm in width are possible at sample flow rates of 2.4 ml/min, with thickness varying from 1.5 *μ*m to 600 nm. The larger sheet size allows the use of higher laser power than was tolerated by the gas-accelerated chips, as the interaction region is further from the nozzle. These converging nozzles will be detailed in an upcoming publication.

### D. Noise levels

**FIG. 6.**
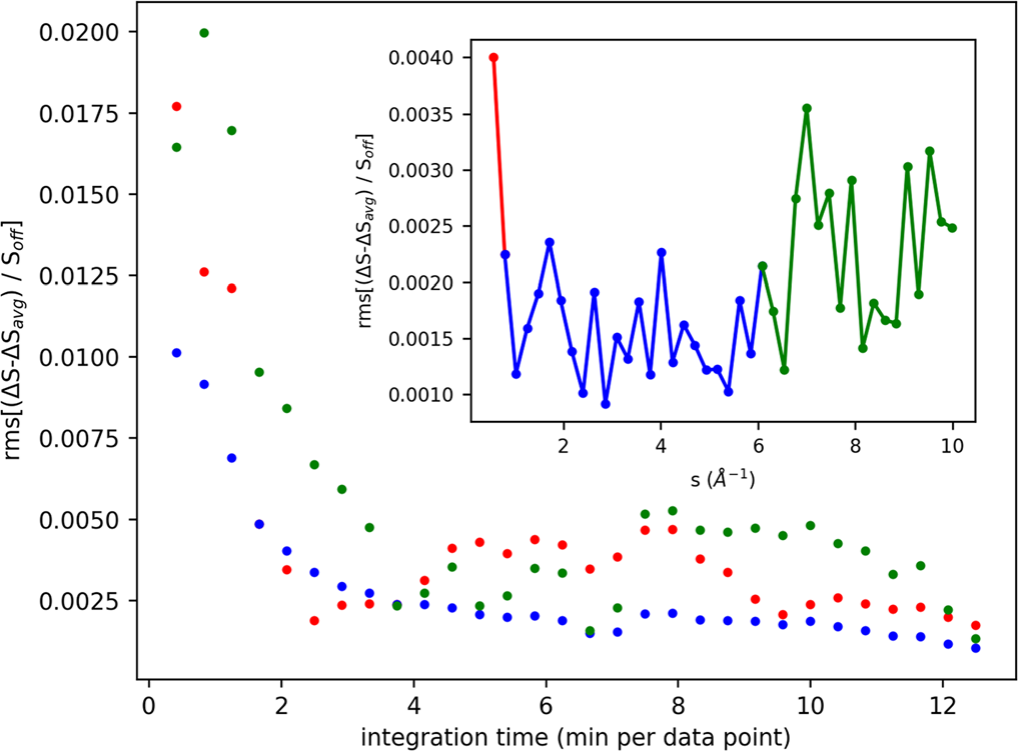
Noise levels of difference scattering from pure water pumped with 3 *μ*m light at 2 ps time delay. Inset: rms noise after 7 min of integration as a function of *s*. Main figure: noise in range *s *=* *0–0.75 Å^−1^ (red), *s *=* *0.75–6 Å^−1^ (blue), and *s *=* *6–10 Å^−1^ (green) as a function of integration time.

Noise levels for a representative time-resolved LUED experiment are shown in Fig. 6. A pure water jet was pumped with 3 *μ*m light, and images were taken at several time delays. Noise is estimated from the azimuthally integrated difference scattering by subtracting a smoothed average difference signal, normalizing by the total laser-off scattering, and calculating the rms of the remaining noise. The noise estimate was applied to difference scattering measured at a fixed time delay for each of 30 scans. Each scan comprises five images, each integrated for 5 s (1800 shots per image).

Noise below 0.2% rms in the 0.75–6 Å^−1^ range is achieved after 7 min of integration time per pump-probe delay. However, noise in the high-*s* region (> 6 Å^−1^) is consistently higher and exhibits a jump after 7 min. This could be related to fluctuations in the thickness of the jet, which affects the ratio between low- and high-*s* scattering (as discussed in Sec. II C). The noise at the lowest *s* values, below 0.75 Å^−1^, is also higher, despite this being the area with the most counts. Small fluctuations in beam pointing, possibly due to charging effects, could contribute to the noise in this region.

For experiments on pure liquids, difference signals in the percent range are possible, making these types of experiments feasible with 4–5 min of integration per time point. However, scattering from solution samples is dominated by the solvent, generally with thousands of solvent molecules per solute molecule of interest. As a consequence, the difference signals for solutes are typically very small, well below 0.1% compared to total scattering.^43–45^

## III. OUTLOOK

The LUED instrument has extended the UED technique to liquid-phase samples. An ultrathin liquid sheet jet, running in vacuum in the low 10^−4 ^Torr regime, was demonstrated in water, concentrated (hundreds of mM) aqueous solutions of ionic solutes and ethanol. Static scattering from liquid water achieved structural sensitivity over a range of 10 Å with a resolution of 0.6 Å, and structural features associated with hydrogen bonding were observed. The instrument achieved a time resolution of 209 fs FWHM in pump-probe experiments at 30° pump incidence.

Time-resolved experiments targeting changes in the overall structure of pure liquids are already achievable with current signal-to-noise ratios, which allow measurement of difference scattering on the percent level within several hours of data collection. However, challenges remain in studying molecules in solution, due to the large background caused by solvent molecules. Typically, difference signals for structural changes of ∼0.2 Å in solutes at 20–70 mM concentration are below 0.1%.^43^ Several future improvements described below will facilitate these lower signal experiments.

Improvements to signal-to-noise are possible through several system upgrades. Increasing the electron flux by running at a higher repetition rate will decrease required averaging times, with a repetition rate of 1 kHz planned for the SLAC MeV UED facility. Furthermore, a planned upgrade to a direct electron detector,^46^ capable of single-electron detection, will also improve signal-to-noise. The direct detector will enable an electron-counting detection scheme that can potentially eliminate camera readout noise. In addition, the current system requires averaging of many shots into a single image, and saturation prevents imaging of the direct beam at the same time as the scattering pattern. The direct detector will allow imaging of the beam concurrently with the scattering pattern on a shot-by-shot basis. The noise observed in the lowest *s* values with the current detector points to a strong sensitivity to small changes in beam pointing, which can wash out difference signals when many shots are averaged together. With the single-shot detection scheme, beam pointing and proper normalization can be accounted for in each image. In addition, jet stability can be improved by replacing pressure control of the accelerating gas with a mass flow controller, which could eliminate the slow change in jet thickness observed in the electron transmission.

The first experimental run has produced time-resolved experiments on pure water in several excitation regimes as well as observation of dissociation of $I_{3}^{-}$ in solution, which will be reported in upcoming publications. The improvements detailed above will extend the potential of the LUED technique from highly concentrated systems (such as neat solvent) to more dilute chemical samples. Future photochemical experiments have the potential to exploit the Coulomb potential sensitivity of electrons to act as a complementary method to x rays, especially to observe reactions involving proton transfer.

## IV. METHODS

### A. Integration with the SLAC MeV UED beamline

**FIG. 7.**
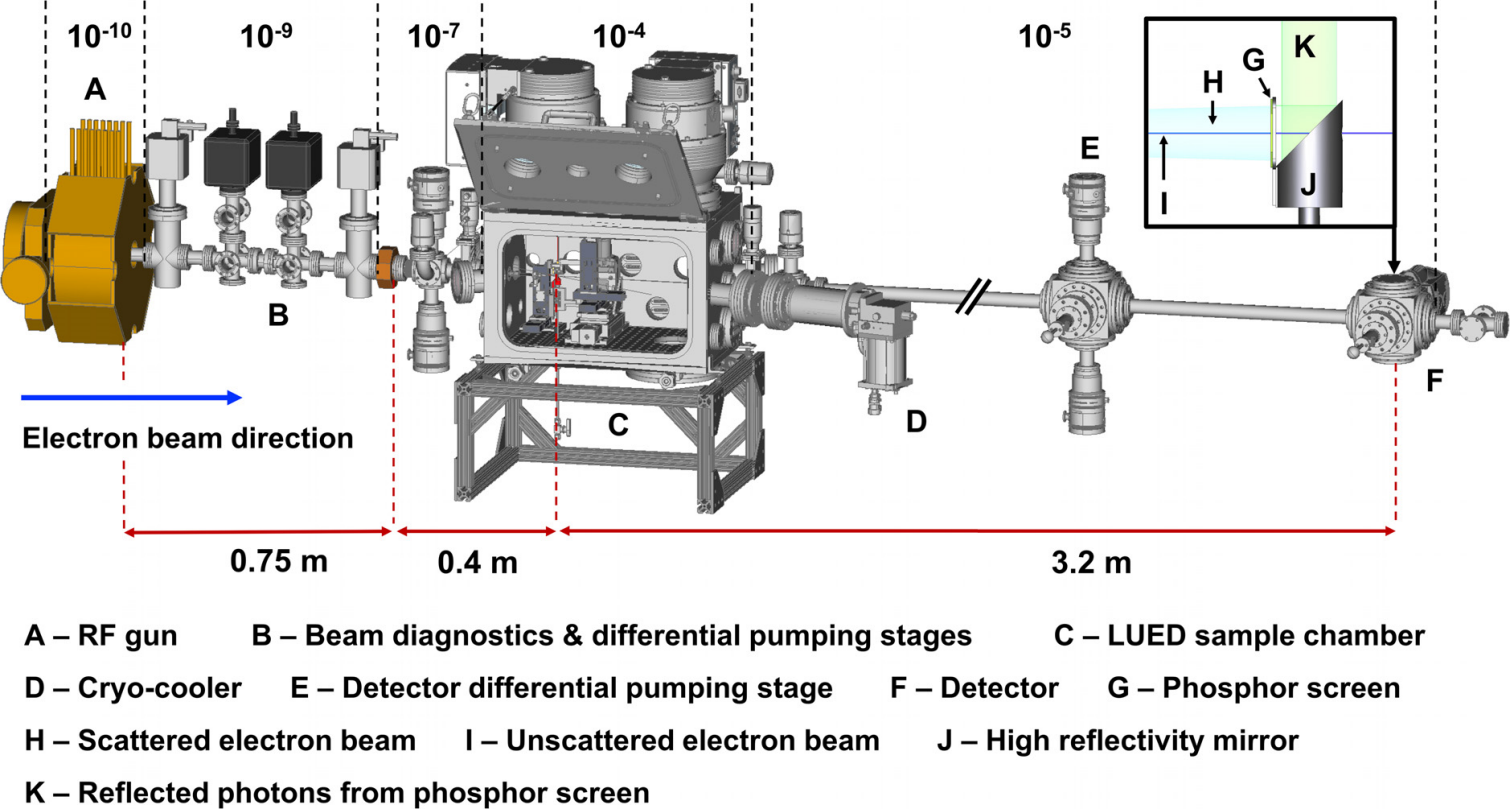
3D CAD model of the SLAC MeV beamline and LUED sample chamber. Typical operating pressure of various differentially pumped sections is presented in Torr above the beamline. The inset on the top right corner illustrates the geometry of the detector.

The LUED sample chamber was installed 0.75 m downstream of the photocathode RF gun in the SLAC MeV UED beamline, schematically depicted in Fig. 7 and described in further detail elsewhere.^47^ The 3.7 MeV electron beam produced by the S-band 1.6-cell photocathode RF gun is delivered to the interaction point, 1.15 m downstream, with a spot size of 88 × 37 *μ*m^2^ FWHM at an average bunch charge of 2 fC. Higher bunch charges (up to 100 fC) are available at the expense of temporal resolution, spot size, and reciprocal-space resolution. The system operates at a repetition rate up to 360 Hz; in this work, the repetition rate was 180 Hz for data shown in Figs. 2, 4, and 5, and 360 Hz for data shown in Figs. 3 and 6. A series of differential pumping stages with elongated chokes decouple the vacuum in the RF gun (10^−10 ^Torr) from that of the sample chamber (10^−4 ^Torr). Located 3.2 m downstream of the interaction region, the SLAC MeV UED detector consists of an in-vacuum back-illuminated phosphor screen and high-reflectivity mirror, oriented 90° and 45° with respect to the beam path, respectively. A schematic representation of the detector geometry is shown as an inset in Fig. 7. A hole through the center of the phosphor screen (3 mm dia.) and mirror assembly (4.5 mm dia.) allows the unscattered electrons to pass through the detector, improving the detector dynamic range and preventing saturation. Photons generated by the incidence of a scattered electron on the phosphor screen are coupled out of the beamline and collected by a 50 mm f/1.2 lens onto an Andor iXon Ultra 888 electron-multiplying charge-coupled device (EMCCD). The SLAC MeV UED beamline is driven by a Ti:sapphire laser system, producing 65-fs 800 nm laser pulses at a repetition rate of 360 Hz. A small portion of this laser light (400 *μ*J) is frequency tripled to 266 nm and used in the generation of photoelectrons at the RF gun, with the remaining (>12 mJ) made available to the optical excitation of samples at wavelengths ranging from 240 to 2400 nm accessible via harmonic generation or optical parametric amplification. The delivery of optical pump pulses to the interaction point is discussed in Sec. IV B 2.

### B. Sample chamber

The LUED sample chamber was constructed to house a variant of the ultrathin free-flowing liquid sheet sample delivery system developed by Koralek *et al.* and described elsewhere.^15^ In brief terms, a sub-micrometer free-flowing liquid sheet is formed by the flattening and shaping of a cylindrical liquid jet by two converging gas jets. The liquid and gas jets are delivered to vacuum using a three-channel borosilicate microfluidic chip (Micronit). The resulting liquid sheet is collected a few millimeters below the chip. A more detailed description of this sample delivery system can be found in Sec. IV C. The LUED sample chamber design fulfills five major requirements: maintain three orders of magnitude pressure differential between the sample chamber and the in-coupling mirror, allow near-collinear pump and probe of the target sample, allow remote alignment of the chip and catcher assembly, accommodate an interferometric sheet thickness measurement tool, and allow for quick and unrestricted access to the interaction region. Design features addressing these requirements are presented in Secs. IV B 1–IV B 5.

#### 1. Vacuum system

The gas load associated with using a free-flowing liquid sheet can be divided into two main contributions: the continuous flow of gas used to flatten the otherwise cylindrical liquid jet into a thin sheet and the gas load associated with the evaporation of liquid sample. The magnitude of the latter is strongly dependent on the vapor pressure of the sample. Our liquid sheets are shaped using helium, the elastic scattering cross sections of which is small enough so as not to contribute significantly to the diffraction signal. In the LUED chamber, the helium and sample gas loads are managed using two vertically mounted 1300 Ls^−1^ turbo molecular pumps. Additionally, a high surface area cold trap cryo-cooled to 70 K can also be used to help manage the gas load associated with sample evaporation. Under typical flow-rates of 0.25 ml/min of liquid sample and 100 sccm of helium, chamber pressures of 10^−4 ^Torr can be maintained for more than 24 h. Pressures here reported were achieved without the use of the cold-trap and thus represent the upper limit of our operating conditions. A differential pumping stage fitted with two 30 Ls^−1^ turbo molecular pumps and a protruding capillary maintains up to three orders of magnitude pressure differential between the sample chamber and the MeV beamline. This differential pumping stage, henceforth referred to as the incoupling cube, is also responsible for housing and preventing the chemical contamination of optics used in the incoupling of quasi-collinear pump pulses. The two differential pumping turbo molecular pumps in the incoupling cube are backed by an 80 Ls^−1^ turbo molecular pumping station, thus ensuring the compression ratios necessary for the pumping of helium. In the case of pressure spikes, adequate vacuum isolation between the MeV beamline and the sample chamber is ensured by two gate valves installed on either side of the chamber and interlocked to the beamline vacuum gauges. Typical pressures for the chamber, in-coupling cube, MeV beamline, and RF gun are presented in Fig. 7.

#### 2. Incoupling optics

**FIG. 8.**
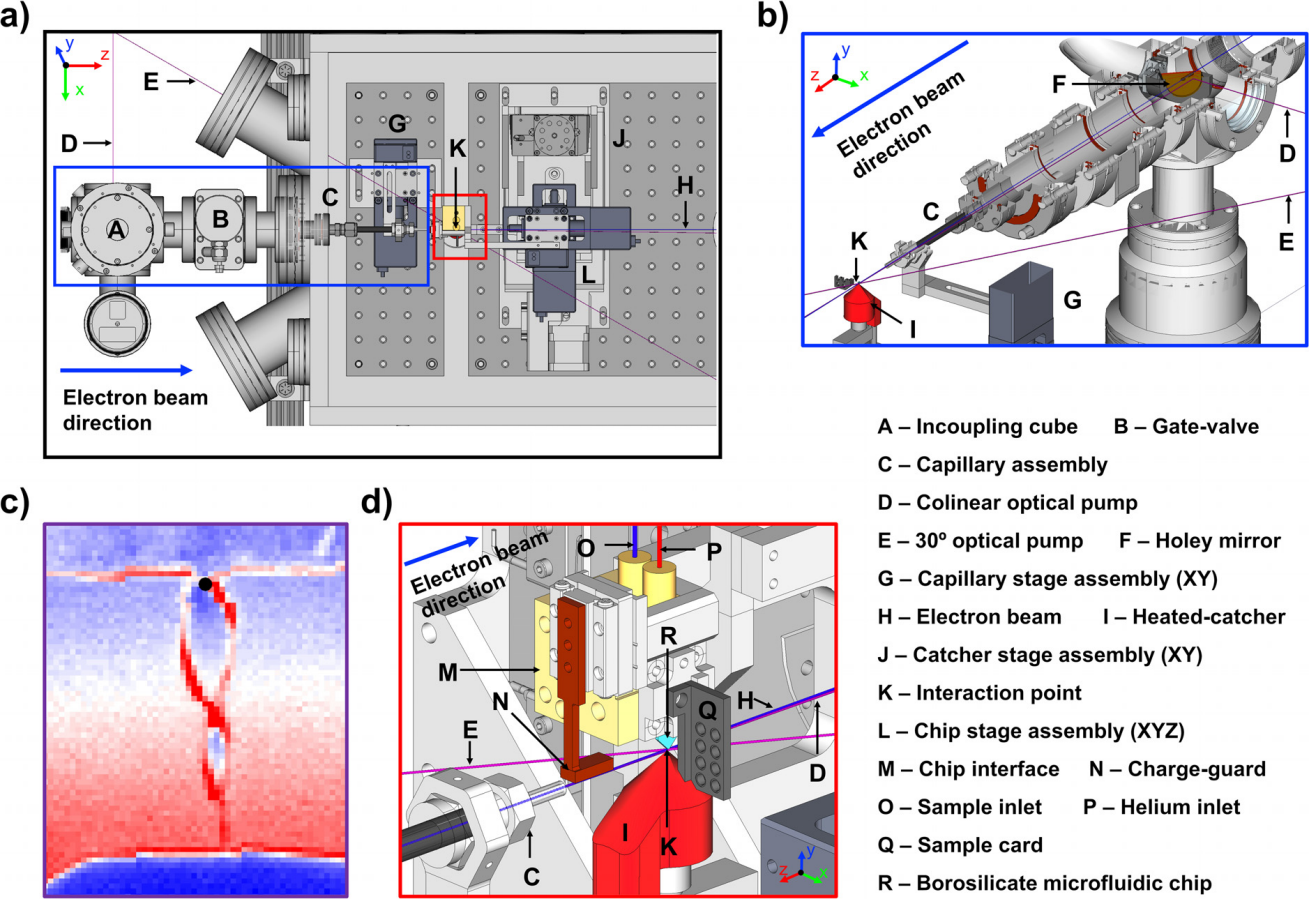
(a) CAD model of the inside of the SLAC MeV LUED chamber. (b) CAD model of the incoupling mirror and capillary assembly. (c) Photography of the liquid sheet in false color. (d) CAD model depiction of the interaction region geometry. The chamber walls are omitted for visualization purposes.

The LUED setup was designed to accommodate three optical pumping geometries: quasi-collinear, 15° counter-propagating, and 30° co-propagating with respect to the electron beam path. In its collinear configuration, the pump laser is incoupled through an in-vacuum 90° holey-mirror positioned inside the incoupling cube stage, and delivered to the interaction point via a long capillary. A 4 mm thick copper shower-stopper protects the mirror substrate from potentially damaging stray MeV electrons. The position of the capillary can be adjusted remotely in two degrees of freedom [see Fig. 8(b)], which facilitates the overlap between pump and probe beams and allows adequate clearances to be maintained between the beams and the inner walls of the capillary. The length and internal diameter of the capillary can be adjusted depending on the vacuum requirements and vapor pressure of the sample, with typical dimensions ranging between 10 and 30 mm in length and 1.2–2.5 mm in internal diameter. Geometric constraints imposed by the capillary, as well as the damage threshold of the incoupling mirror, limit the collinear optical pump energy and spot size to 200 *μ*J and 300 *μ*m, respectively. In experiments where higher pump fluences are required, such as studies on warm dense matter^48^ or strong field ionization, two 4.5 in. conflat windows oriented at 15° and 30° with respect to the interaction region are available. These optical pump geometries allow access to high pump fluences, albeit at the cost of temporal resolution (see Sec. II B).

#### 3. In-vacuum manipulation

The position of the borosilicate microfluidic chip with respect to the heated-catcher is controlled by three vacuum stages in an xyz configuration. Two cameras positioned 30° and 90° with respect to the electron beam path provide real-time feedback on the positions of the chip and catcher, allowing for remote operation and alignment. A PEEK (polyether ether ketone) interface that holds the chip also mounts a copper charge-guard that protects the chip from charging by stray MeV electrons. The charge-guard is lowered towards path of the electron beam using a piezo-vacuum-stage. A diagnostic paddle containing a YAG screen and several crystalline samples is mounted onto the chip clamp with its sample plane matching that of the liquid sheet. This paddle is used to optimize the spatial overlap between the pump and probe beams, as well as provide a rough estimate of the time-zero position based on the profile of the Debye Waller response of photo-excited crystalline samples. The chip assembly and catcher are mounted on an xy-stage assembly, thus allowing the diagnostic paddle to be moved into the path of the electron beam, while maintaining the alignment between the catcher and chip. A CAD (computer-aided design) model illustrating the layout of vacuum stages and geometry of the interaction region is shown in Fig. 8(a).

#### 4. Interferometric sheet thickness measurement

The thickness of a free-flowing liquid sheet can be estimated from the position and number of thin-film interference fringes observed when a sheet is illuminated with a monochromatic source of a known wavelength.^49^ The condition for constructive interference is

$$2n_{\mathrm{film}}d\;\text{cos}\;(\theta )=\left(m-\frac{1}{2}\right)\lambda ,$$ (9)

where *n_film_* and *d* are the refractive index and thickness of the sheet, respectively, *λ* is the wavelength of the monochromatic source in the liquid medium, *θ* is the angle of reflection in the interior of the film, and *m* is an integer. Inside the LUED sample chamber, the free-flowing liquid sheet was illuminated with 505 nm light from a light-emitting diode (LED) device, mounted on a window upstream of the interaction region and angled 30° from normal with respect to the liquid sheet. The resulting thin-film interference fringes are recorded using a camera mounted at a 60° with respect to the light source. The result of these thickness measurements is presented in Fig. 4.

#### 5. Accessibility

The LUED chamber has a 24 × 16 in. hinged door allowing nearly unrestricted access to the inside of the chamber. The large access door facilitates the servicing and replacement of microfluidic chips. A 12 in. gap underneath the chamber allows access to the sample collection bottle, valves, and cooling system associated with the heated catcher system described in Sec. IV C.

### C. Sample delivery

The microfluidic chip [R in Fig. 8(d)] is held in a PEEK interface (M), which connects to liquid (O) and gas (P) lines. The gas line is fed 0–150 psi helium via a remote-controlled pressure regulator. The liquid line connects to a multi-position valve actuator to allow switching between several inputs. The main (sample) input is fed by a high-performance liquid chromatography (HPLC) pump, which can deliver stable liquid flow at the typical flow rates of 0.1–0.4 ml/min. Pure solvent is delivered through a second input from a pressurized steel bottle, with flow controlled by a second remote pressure regulator. The gas and liquid are filtered through 5 *μ*m frits to prevent chip clogging.

A heated catcher [Innovative Research Solutions, I in Fig. 8(d)] is positioned close to the tip of the chip and captures the jet after less than 1 mm of in-vacuum flow, to control the pressure in the sample chamber and to allow reuse of the sample. The catcher consists of a hollow copper–beryllium cone, heated to 100 °C, with a 500 *μ*m hole to capture the jet. Below the cone is a flexible tube which leaves the vacuum chamber through a feedthrough and ends in a collection bottle below the chamber. This bottle is kept under vacuum by a chemical-resistant membrane pump. To prevent evaporation of the captured sample, the collection bottle is submerged in a chilled bath kept at a temperature where the vapor pressure of the solvent is below 10 mbar. The collection bottle can be valved off from the chamber and emptied without venting the sample chamber, allowing longer running times between venting.

Freezing of the jet, often an issue in liquid-phase experiments under vacuum, was mitigated by the heated catcher. In-vacuum start and stop operation of the jet, without venting the sample chamber was possible. The main failure modes of the jet were clogging, mitigated by filters, and laser damage, which set an upper limit to the laser power available to gas-accelerated jet experiments. Incidence of laser pulses with fluence exceeding 1.4 J/cm^2^ at 800 nm onto jets 200–300 *μ*m below the chip caused reproducible and non-reversible failure of the jet.

## AUTHOR'S CONTRIBUTIONS

J.P.F.N. and K.L. contributed equally to this work. J.P.F.N. designed the LUED sample chamber and analyzed the static water data; K.L. designed the sample delivery system and analyzed the time-dependent diffraction data; J.P.F.N. and K.L. wrote the manuscript; M.L. performed time-resolved LUED measurements of water pumped with 800 nm light; D.P.D. developed the gas-accelerated and converging liquid jets and supervised their adaptation to UED; E.B. and Y.L. acquired LUED data; M.C. supervised the analysis of LUED data and advised the writing of the manuscript; C.C. participated in adapting converging liquid jets to UED; M.D. developed the control system and data acquisition tools used in the LUED experiments; S.G. and R.S. advised the design of the LUED sample chamber and sample delivery system, respectively; M.M. performed LUED experiments using converging nozzles; X.S. optimized the MeV electron beam for LUED experiments; K.J., S.W., and C.Y. supervised the commissioning of the LUED sample chamber; T.J.A.W., J.Y., and A.A.C. performed LUED experiments, advised on the design and implementation of the LUED chamber and sample delivery, and advised the writing of the manuscript; X.J.W. supervised the project.
